# Dynamic MAIT Cell Recovery after Severe COVID-19 Is Transient with Signs of Heterogeneous Functional Anomalies

**DOI:** 10.4049/jimmunol.2300639

**Published:** 2023-12-20

**Authors:** Tobias Kammann, Jean-Baptiste Gorin, Tiphaine Parrot, Yu Gao, Andrea Ponzetta, Johanna Emgård, Kimia T. Maleki, Takuya Sekine, Olga Rivera-Ballesteros, Karolinska COVID-19 Study Group, Sara Gredmark-Russ, Olav Rooyackers, Magdalena Skagerberg, Lars I. Eriksson, Anna Norrby-Teglund, Jeffrey Y.W. Mak, David P. Fairlie, Niklas K. Björkström, Jonas Klingström, Hans-Gustaf Ljunggren, Soo Aleman, Marcus Buggert, Kristoffer Strålin, Johan K. Sandberg

**Affiliations:** * Center for Infectious Medicine, Department of Medicine Huddinge, Karolinska Institutet, Karolinska University Hospital, Stockholm, Sweden; † Department of Infectious Diseases, Karolinska University Hospital, Stockholm, Sweden; ‡ Department of Clinical Interventions and Technology, Karolinska Institutet, Stockholm, Sweden; § Perioperative Medicine and Intensive Care, Karolinska University Hospital, Stockholm, Sweden; ¶ Department of Physiology and Pharmacology, Karolinska Institutet, Stockholm, Sweden; ǁ Institute for Molecular Bioscience, The University of Queensland, Brisbane, Queensland, Australia; # Division of Infectious Diseases and Dermatology, Department of Medicine Huddinge, Karolinska Institutet, Stockholm, Sweden

## Abstract

Mucosal-associated invariant T (MAIT) cells are an abundant population of unconventional T cells in humans and play important roles in immune defense against microbial infections. Severe COVID-19 is associated with strong activation of MAIT cells and loss of these cells from circulation. In the present study, we investigated the capacity of MAIT cells to recover after severe COVID-19. In longitudinal paired analysis, MAIT cells initially rebounded numerically and phenotypically in most patients at 4 mo postrelease from the hospital. However, the rebounding MAIT cells displayed signs of persistent activation with elevated expression of CD69, CD38, and HLA-DR. Although MAIT cell function was restored in many patients, a subgroup displayed a predominantly PD-1^high^ functionally impaired MAIT cell pool. This profile was associated with poor expression of IFN-γ and granzyme B in response to IL-12 + L-18 and low levels of polyfunctionality. Unexpectedly, although the overall T cell counts recovered, normalization of the MAIT cell pool failed at 9-mo follow-up, with a clear decline in MAIT cell numbers and a further increase in PD-1 levels. Together, these results indicate an initial transient period of inconsistent recovery of MAIT cells that is not sustained and eventually fails. Persisting MAIT cell impairment in previously hospitalized patients with COVID-19 may have consequences for antimicrobial immunity and inflammation and could potentially contribute to post-COVID-19 health problems.

## Key Points

- Transient numerical MAIT cell recovery after severe COVID-19 eventually fails.
- Temporarily recovering MAIT cells display heterogeneous functional impairment.
- Decline in MAIT cell numbers and function associated with a PD-1^high^ profile.

## Introduction

Severe acute respiratory syndrome coronavirus 2 is the airborne agent causing COVID-19 and has caused millions of deaths worldwide since its appearance in late 2019. COVID-19 is characterized by a complex interplay between innate and adaptive immune mechanisms, host genetics, and preexisting conditions, generating a wide range of disease severity from asymptomatic to critical acute respiratory distress (1, 2). Long-term symptoms, collectively termed “postacute sequelae of COVID-19” (PASC), also known as post-COVID or long COVID, occur in a minority of convalescent individuals with a range of systemic, cardiorespiratory, neuropsychiatric, and gastrointestinal manifestations (1, 3, 4).

SARS-CoV-2 infection provokes a rapid innate immune response that is often successful and followed by an adaptive immune response that clears the infection (1, 5). However, in some individuals, an imbalanced and excessive innate response drives progression to excessive inflammation, coagulopathy, and organ damage (2). The imbalanced type of innate response involves an impaired and delayed type I IFN response (6–8), coupled with excessive activation of the myeloid compartment (6, 9). Furthermore, severe COVID-19 is also associated with excessive activation of an abundant population of unconventional T cells with innate-like response characteristics known as mucosal-associated invariant T (MAIT) cells (10–12). The MAIT cell population expresses a semi-invariant TCR repertoire and primarily recognizes microbial vitamin B_2_ metabolites, such as the strong agonist 5-(2-oxopropylideneamino)-6-*d*-ribitylaminouracil (5-OP-RU), presented by the nonpolymorphic and evolutionarily conserved MHC class I–related protein 1 (MR1) molecules (13–16). MAIT cells play an important role in barrier immunity (17, 18), and murine models support their important role in the control of bacterial infections in the lung (19–21). MR1-restricted responses of MAIT cells include rapid production of diverse cytokines such as IFN-γ, TNF, IL-17, and IL-22 (22, 23), as well as cytolytic effector function against infected cells (24, 25). Furthermore, recent evidence indicates that MAIT cells can participate in MR1-dependent tissue repair (26–28).

Beyond their role in MR1-restricted antibacterial immunity, it is also clear that MAIT cells respond strongly to some viral infections (29–32). This includes a protective effect against influenza virus infection, which is probably mediated via IFN-γ production (33, 34). Antiviral responses by MAIT cells are driven primarily by innate cytokines produced in response to virus, including IL-12, IL-15, IL-18, and IFN-α (35, 36). SARS-CoV-2 infection provokes strong activation of the MAIT cell pool in humans and leads to a decline in circulating MAIT cell numbers in peripheral blood (10–12). Strikingly, in severe COVID-19, the MAIT cell activation levels are associated with and predictive of mortality (10, 11, 37). However, little is known regarding the ability of the MAIT cell compartment to recover in hospitalized patients who survive the acute stage of severe disease. In the present study, we have addressed this by longitudinal sampling of patients with COVID-19 up to 9 mo after release from the hospital. The findings indicate an initial transient period of inconsistent recovery of the MAIT cell compartment that is not sustained and ultimately fails. Because MAIT cells have important roles in antimicrobial immunity and inflammation, we hypothesize that long-term impairment of these cells may play a role in persistent health problems in previously hospitalized patients with COVID-19.

## Materials and Methods

### Patient characteristics

Twenty-seven patients with SARS-CoV-2 infection hospitalized with moderate-severe and severe COVID-19 were sampled in April and May 2020 as part of the Karolinska KI/K COVID-19 Immune Atlas project (10, 38–40). Twenty-four patients were available for studies of MAIT cells at the acute stage of the disease. Detailed demographics and clinical characteristics of the acute cohort were previously described (10). The 20 patients who survived the acute phase of COVID-19 and were released from the hospital were followed over time. Blood samples were collected 4 and 9 mo after acute disease resolution from 16 and 13 patients, respectively (Supplemental Table I). Eleven age- and sex-matched SARS-CoV-2 IgG seronegative control subjects were sampled during the fall of 2020. PBMCs from patients and control subjects were isolated, cryopreserved, and thawed at the same time for analysis. Cryopreserved samples from a second cross-sectional group of 24 hospitalized patients with COVID-19 sampled at 4–5 mo of convalescence were subsequently identified from the Karolinska KI/K COVID-19 biobank to extend the size of the cross-sectional study cohort (Supplemental Table II). All blood donors gave written informed consent in accordance with study protocols conforming to the provisions of the Declaration of Helsinki and approved by the regional ethics review board in Stockholm.

### Absolute blood cell counts

Absolute cell counts in whole blood were assessed by flow cytometry using BD Multitest 6-color TBNK reagents in association with BD Trucount tubes (BD Biosciences) following the manufacturer’s instructions. CD3^+^ T cells were gated from CD45^+^ CD14^−^, CD15^−^, and CD19^−^ cells, and the number of events obtained was used to determine the absolute CD3 counts as follows: (number of CD3^+^ events acquired × number of beads per tubes)/(number of beads events acquired × sample volume in μl). The absolute count of MAIT cells analyzed was subsequently calculated using their percentages out of total CD3^+^ T cells.

### Flow cytometry staining procedure and Abs

The following Abs were used for staining: CD69-BUV395 (clone FN50), CD38-BUV496 (clone HIT2), CD56-BUV737 (clone NCAM16.2), CD3-BUV805 (clone UCHT1), CD14-V500 (clone M5E2), CD19-V500 (clone HIB19), iNKT-BV750 (clone 6B11), CD161-PE-Cy5 (clone DX12), granzyme A (GzmA)-PerCP-Cy5 (clone CB9), GzmB-AF700 (clone GB11), CD107a-BUV395 (clone H4A3), and TNF-PE-Cy7 (clone Mab11) from BD Biosciences; PD1-BV421 (clone EH12.2H7), CD8-BV570 (clone RPA-T8), IL-7R-BV605 (clone A019D5), CD4-BV711 (clone OKT4), HLA-DR-BV785 (clone L243), Ki67-AF488, V*a*7.2-PE-Cy7 (clone 3C10), CXCR6-AF647 (clone K041E5), IL-17A-BV421 (clone BC168), CD3-BV650 (clone OKT3), GzmB-FITC (clone GB11), TCRγδ-PE-Dazzle594 (clone B1), and V*a*7.2-PE (clone 3C10) from BioLegend. PE- and allophycocyanin-conjugated 5-OP-RU–loaded human MR1 tetramer (NIH Tetramer Core Facility) was used to identify MAIT cells. The LIVE/DEAD Fixable Near-IR Dead Cell Stain Kit (Thermo Fisher) was used for staining dead cells. PBMCs were first stained with the hMR1-5-OP-RU tetramer for 30 min at room temperature prior to extracellular staining for another 20 min at 4°C in PBS 2 mM EDTA and 2% FBS. After washing, cells were fixed and permeabilized in BD Cytofix/Cytoperm Fixation/Permeabilization Kit (BD Biosciences) for 30 min at 4°C and stained intracellularly in 1× BD Perm/Wash buffer (BD Biosciences) for 30 min at 4°C. After washing, cells were fixed for 10 min at room temperature in BD CellFIX 1× (BD Biosciences) before acquisition. Samples were acquired on a BD FACSymphony A5 flow cytometer (BD Biosciences) and analyzed with FlowJo software version 10.7.1 (BD Biosciences).

### Functional assays

For cytokine stimulation, PBMCs were cultured for 24 h in complete media (RPMI 1640 medium supplemented with 10% FCS, 2 mM l-glutamine, 100 U/ml penicillin, and 100 μg/ml streptomycin) with IL-12p70 (10 ng/ml; PeproTech) and IL-18 (100 ng/ml; MBL international) in the presence of CD107a Ab. Monensin (GolgiStop; BD) and brefeldin A (GolgiPlug; BD) were added for the last 6 h before staining. For antigenic stimulation, PBMC were incubated for 2 h with 100 nM 5-OP-RU in the presence of CD107a Ab before adding monensin and brefeldin A for 6 h. The MAIT cell Ag 5-OP-RU was synthesized as a DMSO solution, in which it remains chemically stable and intact, as previously described (16, 41).

### Flow cytometric analysis and Uniform Manifold Approximation and Projection

Single-stained compensation beads (BD Biosciences) were used to calculate the compensation matrix. FCS3.0 files exported from BD FACSDiva software were imported into FlowJo software, and a compensation matrix was generated using the AutoSpill/AutoSpread algorithm. Data were cleaned prior to analysis using the FlowAI algorithm that checks the stability of the flow over time for each of the parameters acquired. The resulting good events were exported and used for downstream analysis. Patients with fewer than 10 events in the MAIT cell gate were not included in the phenotypic or functional analysis. To generate the Uniform Manifold Approximation and Projection (UMAP), samples were first downsampled using the FlowJo plugin Downsample (version 3.2), barcoded with the patient group, and concatenated. A total of 1000 MAIT cells per patient were included. The FlowJo plugin UMAP (version 3.1) was run on the resulting FCS file using the default settings (Distance function = Euclidean, nearest neighbors: 15 and minimum distance: 0.5), including all the compensated parameters and forward scatter and side scatter measurements.

### Principal component analysis

Principal component analysis (PCA) of MAIT cell phenotypes was performed in R using the FactoMineR package (version 2.8). PCA was computed on the expression levels of CD8, CD38, CD56, CD69, HLA-DR, GzmA, GzmB, PD-1, Ki67, CD161, CXCR6, and IL-7 receptor α subunit (IL-7R, CD127). Median fluorescence intensity measurements for GzmA, PD-1, Ki67, CD161, CXCR6, and IL-7R were batch corrected to the geometric mean fluorescence intensity of each flow cytometry experiment. All parameters were then scaled before PCA computation.

### Combinatorial polyfunctionality analysis of Ag-specific T cell subsets

The combinatorial polyfunctionality analysis of Ag-specific T cell subsets (COMPASS) algorithm creates an unbiased model of observed cell subsets displaying different protein marker combinations and infers Bayesian statistics to present polyfunctional subsets that are likely to occur in cells responding to stimulation in reference to an unstimulated control condition. Subject- and cell population–specific responses were interrogated by calculation of a polyfunctionality score (42). The SimpleCOMPASS implementation was used to infer the diversity of functional responses of MAIT cells after stimulation as indicated. Combinations of IFN-γ, TNF, GzmB, IL-10, IL-17A, and CD107a were included in the COMPASS model. Boolean gates of all effector molecules were created for each donor and MAIT cell population. Cell subset counts were exported from FlowJo, and COMPASS models were calculated for each donor and MAIT cell subset in comparison with unstimulated control cells using the COMPASS package in R (version 1.3.0) with eight replications of 40,000 iterations per model. Heatmaps were modified from COMPASS’s heatmap function and show the posterior probability of response of prioritized subsets. The probability of an at least bifunctional response was calculated using COMPASS’s response function.

### Statistical analysis

Prism version 9.0.1 (GraphPad Software), Python, and R were used for statistical analysis. Longitudinal statistical analysis of incomplete paired data sets was done using mixed-effect analysis. Statistically significant differences (*p* < 0.05) between longitudinal donor sampling between the 4- and 9-mo time points were determined using a donor-paired Wilcoxon signed-rank test. For comparison of more than two groups, the Kruskal-Wallis test followed by Dunn’s post hoc test and Benjamini-Hochberg correction of *p* values was applied. Correlations were evaluated using the Spearman correlation implemented in the hMISC package (version 5.1) in R. Calculation of the coefficient of covariation was derived from quantification of the donor-to-donor expression variation as recently described (43). In brief, the mean expression of each protein across groups was calculated, subject- and protein-specific expression was normalized to the average, and the group-specific SD for each normalized protein expression was calculated.

### Data and material availability

Curated flow cytometry data from the acute time-point are available for exploration via the Karolinska COVID-19 Immune Atlas (https://covid19cellatlas.com/#/). Other data are available upon request from the corresponding author.

## Results

### Initial recovery of MAIT cells in COVID-19 is transient and fails at 9 mo of follow-up

**FIGURE 1.**
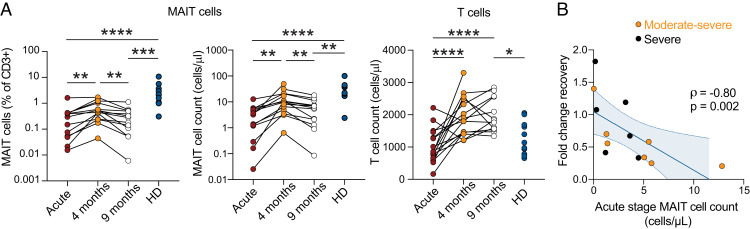
Transient MAIT cell recovery in convalescent COVID-19 patients. (**A**) Relative frequencies of MAIT cells (left), absolute counts of MAIT cells (middle), and absolute counts of T cells (right) in patients with COVID-19 during the acute (*n* = 13) and convalescent phase (*n* = 16 and *n* = 13 for 4-mo and 9-mo time points, respectively) compared with HDs (*n* = 11). Mixed-effect analysis and Kruskal–Wallis tests were used to compare paired and nonpaired data. *p < 0.05, **p < 0.01, ***p < 0.001, ****p < 0.0001. (**B**) Spearman correlation between the absolute count of MAIT cells at the acute phase of COVID-19 infection and the fold change recovery of MAIT cell count between the acute and 4-mo convalescent phase (*n* = 13). The orange color for moderate and black for severe reflect the disease severity defined at the acute stage (10). The Spearman correlation coefficient (rho) and the associated calculated *p* value are indicated on the graph.

During the first wave of SARS-CoV-2 infections in Sweden in the spring of 2020, we studied 24 patients hospitalized with COVID-19 to characterize the involvement of the MAIT cell compartment in severe COVID-19 (10). To investigate the ability of MAIT cells to recover, longitudinal paired blood samples from patients were collected at 4–5 (*n* = 16) and 9 (*n* = 13) mo after disease resolution from the original 2020 patient group who survived the acute stage of COVID-19 and were released from the hospital (Supplemental Table I). MAIT cells were quantified by flow cytometry at the time points of convalescence (Supplemental Fig. 1A) in comparison with a matched healthy donor (HD) control group sampled in parallel at the 4-mo time point (Supplemental Table I) and longitudinally compared with levels observed for the same patients at the acute stage of disease. MAIT cell populations initially recovered by 4 mo postinfection, both in absolute counts and as percentages out of CD3^+^ cells (Fig. 1A). However, this recovery was transient because the MAIT cell percentages and counts declined by the 9-mo time point to levels similar to those during the acute stage of disease (Fig. 1A). This pattern was distinct for MAIT cells, because overall T cell counts recovered and remained elevated above those of the HD group throughout 9 mo of convalescence (Fig. 1A). The numerical recovery of the MAIT cell compartment was dynamic in the sense that the fold change recovery at 4 mo correlated inversely with the acute stage MAIT cell count (rho = −0.80; *p* = 0.002) (Fig. 1B). Thus, longitudinal assessment indicates that the MAIT cell pool shows a pattern distinct from the conventional T cell compartment and only transiently recovers before declining again in patients who were hospitalized during the acute stage of COVID-19.

### Heterogeneous MAIT cell alterations in convalescent COVID-19 patients

**FIGURE 2.**
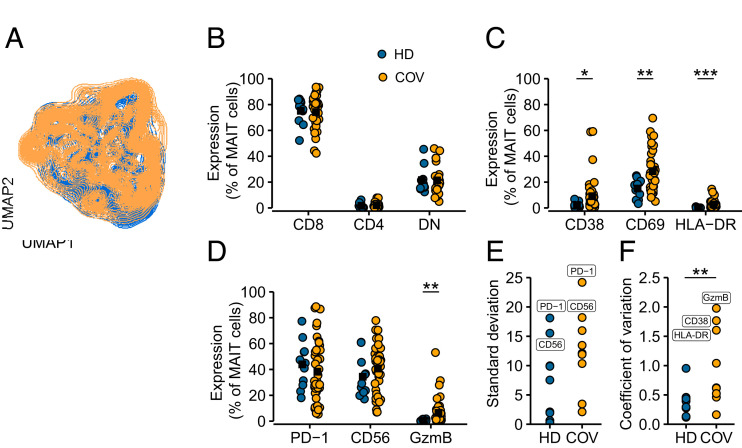
Signs of residual MAIT cell activation in convalescent COVID-19 patients. (**A**) UMAP plots of total live MAIT cells in peripheral blood of HDs (blue) and 4-mo convalescent patients with COVID-19 (COV, orange) colored according to patient group. (**B**) Relative frequencies of MAIT subsets, or (**C**) expression of activation markers, and (**D**) surface receptors of MAIT cells from HDs (*n* = 11) and convalescent patients with COVID-19 (*n* = 41) at resting conditions. Each dot represents one donor, black square indicates group average. The Kruskal–Wallis test followed by Dunn’s post hoc test with *p* value adjustment by Benjamini–Hochberg correction was used to test for statistical differences between groups. *p < 0.05, **p < 0.01, ***p < 0.001. (**E**) SD of phenotypic molecules for each group. (**F**) Coefficient of covariation for each molecule and group. Significance was assessed by Wilcoxon rank-sum test (**p < 0.01).

To better understand the characteristics of the MAIT cell compartment in patients with COVID-19 after recovery from the acute stage of disease, we investigated MAIT cells in PBMC samples from the longitudinal sample set at the 4-mo time point. In addition, we included a second patient group with similar acute disease characteristics, sampled 4–5 mo after release from the hospital (*n* = 24) (Supplemental Table II), to obtain sufficient numbers to investigate phenotypic and functional heterogeneity at this stage of convalescence. MAIT cells were stained for CD4, CD8, CD38, CD56, CD69, HLA-DR, PD-1, Ki67, IL-7R, CXCR6, GzmA, and GzmB and analyzed by flow cytometry (Supplemental Fig. 1B). Dimensionality reduction with UMAP highlighted partially distinct occupation of healthy and convalescent MAIT cell donors on the UMAP topography, indicating alteration of MAIT cell phenotypes by COVID-19 (Fig. 2A). This prompted us to look deeper into the characteristics of the MAIT cell pool at 4 mo of convalescence. Major MAIT cell CD8^+^, CD4^+^, and CD8^−^CD4^−^ double-negative subsets were not perturbed in patients as compared with the HD group (Fig. 2B). However, activation markers such as CD38, CD69, and HLA-DR were elevated (Fig. 2C), and GzmB expression at resting conditions was also significantly higher (Fig. 2D). Expression of PD-1 and CD56, both relevant for MAIT cell effector function, was highly variable in both HD and COVID-19 convalescence groups, as indicated by the largest SDs of all measured proteins (Fig. 2D, 2E). Interdonor variation, quantified by coefficient of covariation, was increased in MAIT cells from convalescent patients with COVID-19 (Fig. 2F). With these patterns at hand, we next investigated in more detail the heterogeneity of MAIT cell compartments in convalescent COVID-19 patients.

**FIGURE 3.**
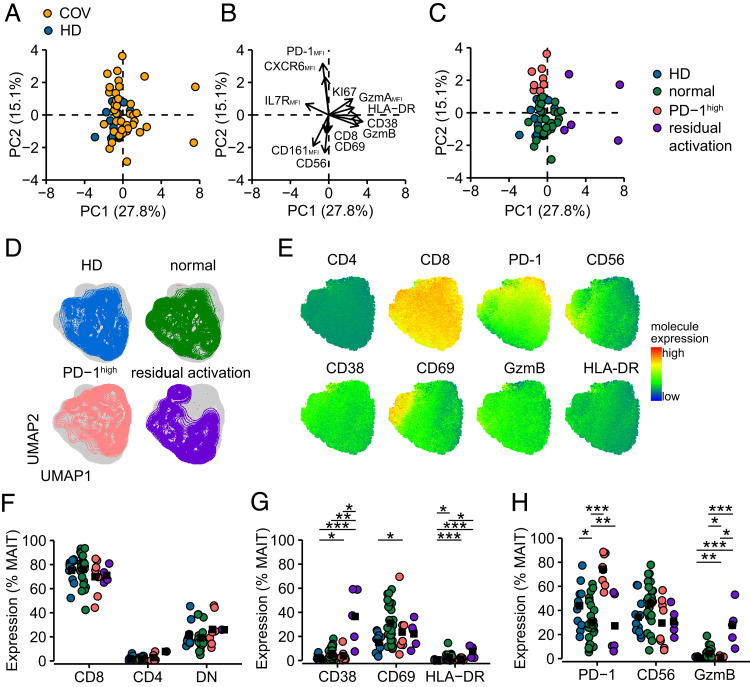
MAIT cell phenotypic heterogeneity in convalescent COVID-19 patients. (**A**) PCA of MAIT cell phenotypic markers of HDs (*n* = 11, blue) and convalescent patients with COVID-19 (COV, *n* = 41, orange). (**B**) Loadings of principal components PC1 and PC2. The arrow length from origin indicates the loading score. (**C**) Convalescent patients were identified to have MAIT cell populations with phenotypic characteristics being normalized (*n* = 28, green), having a PD-1^high^ profile (*n* = 8, red), or residually activated (*n* = 5, purple). (**D**) UMAP plots of MAIT cells from HDs, normalized, PD-1^high^, and residual activated group at resting conditions. (**E**) Expression of molecules visualized on the UMAP of MAIT cells at resting conditions. Color indicates expression value. (**F**) Relative frequencies of MAIT subsets. (**G**) MAIT cell expression of activation markers and (**H**) functionally relevant molecules in samples from HDs or patients with normalized, PD-1^high^, and residually activated MAIT cell pools. Significance of differential protein expression assessed by Kruskal–Wallis test followed by Dunn’s test with Benjamini–Hochberg correction of *p* values (*p < 0.05, **p < 0.01, ***p < 0.001).

PCA indicated that MAIT cells from patients with COVID-19 were phenotypically altered in comparison with the HD group during the transient recovery period at 4 mo of convalescence (Fig. 3A), with phenotypic separation driven by markers of activation along PC1 and PD-1 for PC2 (Fig. 3B). In more detail, this analysis identified three clusters, where the main group of patients exhibited normalized MAIT cell phenotypic patterns overlapping with the HD group, one group segregated from the normalized group based on markers associated with residual activation in MAIT cells, and finally one group characterized by elevated expression of PD-1 (PD-1^high^) (Fig. 3B, 3C). These three subgroups also displayed different topography in the UMAP analysis (Fig. 3D, 3E). Expression of CD8 and CD4 was similar between the groups (Fig. 3F). However, individuals with residually activated MAIT cells expressed increased levels of CD38 (37±23%) and HLA-DR (7.6±4.8%) in comparison with the HD and normalized groups, whereas CD69 levels were similar, a pattern consistent with long-term persistent rather than recent activation (Fig. 3G). Although to a lesser extent, MAIT cells from individuals with the PD-1^high^ MAIT cell characteristics were similarly enriched in activation molecules. However, MAIT cell expression of GzmB at rest was elevated in the residually activated MAIT cell group (28±17%), whereas the opposite pattern was observed in the individuals with PD-1^high^ MAIT cells (0.9±0.8%) (Fig. 3H). These patterns suggest that recovery of the MAIT cell pool in convalescent patients is heterogeneous, and some individuals display altered cellular phenotypes.

### Distinct PD-1^high^ MAIT cell characteristics associated with functional impairment

**FIGURE 4.**
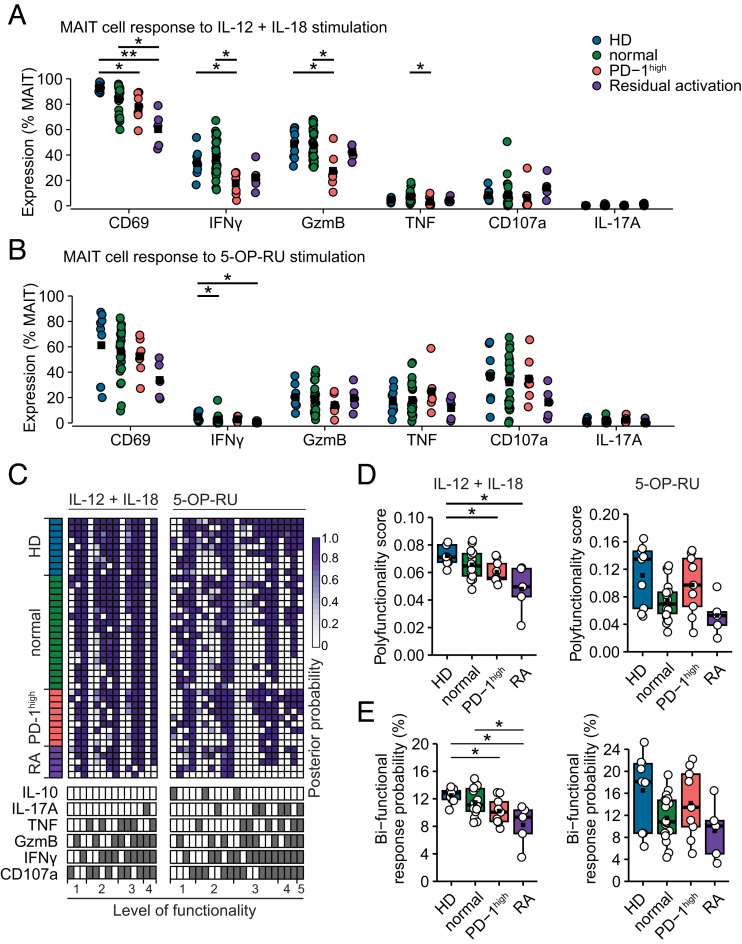
MAIT cell functional heterogeneity in convalescent COVID-19 patients. (**A**) Response of MAIT cells to 24 h IL-12+IL-18 costimulation or (**B**) 5-OP-RU stimulation for 8 h. Colors indicate HDs (*n* = 9) or patient groups with MAIT cell characteristics being normalized (*n* = 26), PD-1^high^ (*n* = 7), or with residual activation (*n* = 5). (**C**) COMPASS heatmap showing posterior probability of response to cytokine (left) or 5-OP-RU stimulation (right). Each row represents one donor. Each column indicates the combination of effector molecules indicated at the bottom of the heatmap. (**D**) Polyfunctionality score calculated by COMPASS for each donor’s response to stimulation for cytokine (left) or 5-OP-RU stimulation (right). (**E**) Probability of response combining two or more effector molecules. Significance was evaluated by Kruskal–Wallis test followed by Dunn’s test with Benjamini–Hochberg adjustment of *p* values (*p < 0.05, **p < 0.01).

MAIT cells respond to TCR recognition of MR1-presented Ag and can also respond, with a limited repertoire of functions, to cytokines produced during viral and other conditions where no MR1-presented Ag is available. To determine if these two modes of activation were affected after the resolution of acute COVID-19, PBMCs collected at the 4-mo follow-up and from HDs were stimulated with either IL-12+IL-18 (Fig. 4A) or MR1-restricted 5-OP-RU Ag (Fig. 4B) (Supplemental Fig. 2A, 2B). Both modes of stimuli yielded strong responses with upregulation of CD69. Response profiles, as expected, differed between stimuli where IL-12+IL-18 promoted a response dominated by IFN-γ and GzmB, whereas the 5-OP-RU–specific response was broader and included CD107a and TNF (Fig. 4A, 4B). Detailed analysis of the patient subgroups revealed that the group characterized by PD-1^high^ MAIT cells demonstrated a poor IFN-γ and GzmB expression in MAIT cells in response to cytokine-mediated activation (Fig. 4A). The patient group characterized by residual MAIT cell activation closely followed the patterns of the PD-1^high^ group. However, CD69 upregulation was low in this group, together with weak IFN-γ production (Fig. 4A, 4B).

We next addressed the quality of the MAIT cell response using COMPASS. In brief, COMPASS uses flow cytometry data of donor-paired stimulated and unstimulated MAIT cells and creates a Bayesian hierarchical mixture model that enables donor-specific and group-specific prediction of coexpression of effector molecules and scoring of the response quality. After IL-12+IL-18 stimulation, MAIT cells showed coexpression of up to four effector molecules (Fig. 4C). After stimulation with 5-OP-RU, MAIT cells mounted a more polyfunctional response, reaching pentafunctionality in some cases, including also IL-17A or IL-10. A trifunctional response of IL-17A, IFN-γ, and CD107a appeared to be enriched in the PD-1^high^ and residual activation groups. MAIT cells of the PD-1^high^ or residual activation groups showed low response quality when measured by COMPASS’s polyfunctionality score (Fig. 4D). Strikingly, low MAIT cell polyfunctionality of the PD-1^high^ group was only seen in response to the cytokine-mediated mode of activation, because the MAIT cell polyfunctionality score was similar to that seen in HD after antigenic TCR stimulation (Fig. 4D). The functional impairment associated with the PD-1^high^ or residually activated MAIT cell phenotype was visible even on the level of bifunctionality, most clearly in response to cytokine stimulation (Fig. 4E). MAIT cells from HD and patients with a normalized MAIT cell phenotype displayed high posterior probability to mount diverse bifunctional responses, whereas patients with PD-1^high^ or residually activated MAIT cells showed reduced levels of GzmB and CD107a or TNF and IFN-γ combinations (Fig. 4E). Altogether, we identify a pattern of poor responsiveness to cytokine-mediated MAIT cell activation in convalescent COVID-19 patients associated with high MAIT cell PD-1 expression or residual activation.

### MAIT cell PD-1 expression increases into 9 mo of COVID-19 convalescence

**FIGURE 5.**
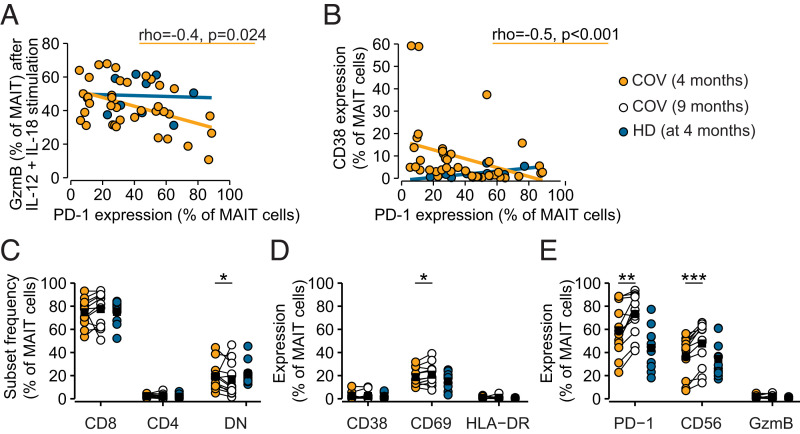
MAIT cell PD-1 expression increases into 9 mo of COVID-19 convalescence. (**A**) Spearman correlation analysis of PD-1 expression on resting MAIT cells against GzmB response to IL-12+IL-18 stimulation or against (**B**) CD38 expression on MAIT cells sampled in 4-mo convalescent patients with COVID-19 (COV, orange) and HDs (blue). Correlation statistics for the COVID-19 convalescent group are indicated. (**C**) Relative frequencies of MAIT cell subsets, or (**D**) expression of activation markers, or (**E**) functionally relevant molecules in MAIT cells from HDs (*n* = 11) and convalescent patients with COVID-19 (*n* = 13) in the longitudinal cohort at 4- and 9-mo follow-up. Significance was evaluated by Kruskal–Wallis test followed by Dunn’s test with Benjamini–Hochberg adjustment of *p* values (*p < 0.05, **p < 0.01, ***p < 0.001).

The large interdonor variability in PD-1 expression (Fig. 2F) prompted us to further investigate associations between PD-1 expression and MAIT cell functionality. MAIT cell PD-1 expression at 4 mo after resolution of acute COVID-19 correlated negatively with GzmB expression following IL-12+IL-18 stimulation, a pattern not replicated in the HD group (Fig. 5A). Furthermore, PD-1 expression correlated inversely with CD38 expression at rest, rendering PD-1 as a predictor of low residual activation in MAIT cells (Fig. 5B).

We next investigated the characteristics of MAIT cells at a later time point, at 9 mo after discharge from COVID-19 hospitalization in the longitudinal cohort, compared with the 4-mo phenotype. MAIT cell CD8, CD4, and double-negative subsets remained largely unaltered at 9 mo of follow-up (Fig. 5C). However, there was a slight increase in CD69 expression (Fig. 5D) and a robust upregulation of CD56 and PD-1 (Fig. 5E). Moreover, MAIT cells from donors who had not returned to normal MAIT cell states and were PD-1^high^ remained less responsive to cytokine stimulation (Supplemental Fig. 2C), as detectable despite reduced sample size for functional analysis. Altogether, these results indicate that some patients recovering from severe COVID-19 have an exhausted MAIT cell compartment characterized by high PD-1 expression and poor responsiveness to cytokine stimulation several months after release from the hospital.

## Discussion

Previous studies by us and others have shown that MAIT cells are strongly affected during severe acute SARS-CoV-2 infection, and findings furthermore suggest that MAIT cell activation and recruitment to the airways may be involved in disease pathogenesis (10, 11, 37). In the present study, we show that patients who were hospitalized with COVID-19 and survived the acute stage of disease mostly recovered their circulating MAIT cell pool when assessed 4–5 mo after release from the hospital. This recovery follows the overall pattern of the total CD3^+^ T cell compartment but is more profound as MAIT cells also increase as a percentage of the total. However, at the 4-mo time point, some patients still have phenotypic and functional anomalies in their MAIT cells, with a subgroup of patients having a PD-1^high^ MAIT cell pool with impaired functionality, possibly reflecting either an overall increase in PD-1 expression or preferential survival of PD-1^high^ MAIT cells in these patients. A second subgroup was rather characterized by residual MAIT cell activation. Unexpectedly, in the patients studied longitudinally, the numerical MAIT cell recovery observed at 4 mo was reversed at 9 mo after release from the hospital, indicating that the dynamic MAIT cell recovery after acute COVID-19 is transient. This pattern was unique to MAIT cells because the total T cell absolute counts stayed elevated at levels above those of the HD control group. Together, these findings indicate an initial transient period of inconsistent recovery of the MAIT cell compartment, which is not sustained and eventually fails.

It has become clear that after resolution of SARS-CoV-2 infection and acute COVID-19, some people experience persistent symptoms of varying type, severity, and persistence (1). Although some of the PASC symptoms are unrelated to the initial disease severity, some long-term effects are more common in patients who were hospitalized at the acute stage (4). Long COVID or post-COVID is a diverse syndrome that can affect a range of organs in various ways beyond the lung pathology observed in severely ill patients. COVID-19, and probably also many aspects of post-COVID, involve persistent inflammation that might be linked to the MAIT cell pattern seen in the present study in peripheral blood, with initial expansion and subsequent decline. We speculate that the initial expansion may involve MAIT cell release from inflamed tissues as the acute stage of disease is cleared. This may mask a more severe impact on the MAIT cell compartment, which becomes evident at the 9-mo follow-up time point, when this effect has subsided, and the remaining inflammation may attract the few remaining MAIT cells. Furthermore, functional and single-cell transcriptomic analyses in patients with COVID-19 recently suggested that MAIT cells are functionally impaired (44) and undergo pyroptosis during the acute stage of the disease (45). This model is also consistent with our observation that some patients have phenotypic and functional anomalies in the MAIT cell pool already at the 4-mo time point.

Findings in other human viral diseases show that the MAIT cell compartment is affected or responds in distinct ways, depending on the type of infection (32). For example, acute HIV-1 infection leads to short-term MAIT cell expansion over the first months (46), whereas chronic HIV infection causes progressive irreversible MAIT cell loss and functional impairment (47, 48). A similar substantial decline of the MAIT cell compartment occurs in chronic viral hepatitis (49). Acute hantavirus infection causes a steep reduction in circulating MAIT cells, which reverses once the virus has been cleared (36). In the present study, we show a more complex pattern after severe COVID-19 with more long-term effects in the MAIT cell compartment, and these may affect immune system performance in the affected host. MAIT cells are important in microbial immune control at barrier tissues such as the lung (17–21) and can also play roles in tissue repair and wound healing. Furthermore, MAIT cells were recently shown to play an unexpected role in the priming of T cell responses to ChAdOx1 encoded vaccine Ags (50) and to be associated with adaptive immune responses to mRNA vaccination (51). Thus, it is possible that persistent MAIT cell impairment after severe COVID-19 may have wide-ranging impacts on immune system performance beyond the classical antibacterial role of MAIT cells.

In this study, we have shown that after severe COVID-19, the MAIT cell compartment goes through an initial transient period of inconsistent recovery, which is not sustained and eventually fails. These (to our knowledge) new insights could be important for interpretation of the roles that unconventional innate-like T cells play in COVID-19 and especially in the long-term effects of this disease. Nevertheless, the study also has limitations. First, the number of patients available for longitudinal sampling was relatively small, thus limiting the ability to detect subgroups of patients. Second, the number of PBMCs available for experiments was limited and thus restricted the range of experiments we were able to perform. Third, the long-term effects of severe COVID-19 on MAIT cells remain uncertain and will need to be examined more comprehensively over time. Fourth, with a small number of patients and a lack of evaluation of PASC symptoms in this group, the current data are only hypothesis generating regarding any MAIT cell involvement in PASC. Finally, the healthy control group was sampled only once at the same time as the 4-mo follow-up of patients with COVID-19. Nevertheless, recent studies indicate that MAIT cell levels in HDs are stable over shorter time periods in the absence of serious inflammatory conditions (51, 52). Given the emerging role of MAIT cells in antimicrobial immunity, inflammation, and tissue homeostasis, we hypothesize that the impairment of these cells may play a role in the persistent health problems experienced by some previously hospitalized patients with COVID-19.

## Karolinska COVID-19 Study Group

Mira Akber^1^, Soo Aleman^2,3^, Lena Berglin^1^, Helena Bergsten^1^, Niklas K. Björkström^1^, Susanna Brighenti^1^, Demi Brownlie^1^, Marcus Buggert^1^, Marta Butrym^1^, Benedict J. Chambers^1^, Puran Chen^1^, Martin Cornillet^1^, Angelica Cuapio^1^, Isabel Diaz Lozano^1^, Lena Dillner^3^, Therese Djärv^4^, Majda Dzidic^1^, Johanna Emgård^1^, Lars I. Eriksson^5^, Malin Flodström-Tullberg^1^, Hedvig Glans^3^, Jean-Baptiste Gorin^1^, Sara Gredmark-Russ^1,3,6^, Jonathan Grip^5^, Quirin Hammer^1^, Alvaro Haroun-Izquierdo^1^, Elisabeth Henriksson^1^, Laura Hertwig^1^, Sadaf Kalsum^1^, Tobias Kammann^1^, Jonas Klingström^1^, Efthymia Kokkinou^1^, Egle Kvedaraite^1,7^, Hans-Gustaf Ljunggren^1^, Marco Giulio Loreti^1^, Magdalini Lourda^1,8^, Kimia T. Maleki^1^, Karl-Johan Malmberg^1^, Nicole Marquardt^1^, Johan Mårtensson^5^, Christopher Maucourant^1^, Jakob Michaëlsson^1^, Jenny Mjösberg^1^, Kirsten Moll^1^, Jagadeeswara Rao Muvva^1^, Pontus Nauclér^3^, Anna Norrby-Teglund^1^, Laura M. Palma Medina^1^, Tiphaine Parrot^1^, André Perez-Potti^1^, Björn P Persson^5^, Lena Radler^1^, Dorota Religa^9^, Emma Ringqvist^1^, Olga Rivera-Ballesteros^1^, Olav Rooyackers^5,10^, Johan K. Sandberg^1^, John Tyler Sandberg^1^, Takuya Sekine^1^, Magdalena Skagerberg^3^, Ebba Sohlberg^1^, Tea Soini^1^, Anders Sönnerborg^2,3^, Kristoffer Strålin^2,3^, Benedikt Strunz^1^, Mattias Svensson^1^, Janne Tynell^1^, Christian Unge^2,4^, Renata Varnaite^1^, Andreas von Kries^1^, David Wullimann^1^

^1^Center for Infectious Medicine, Department of Medicine Huddinge, Karolinska Institutet, Karolinska University Hospital, Stockholm, Sweden. ^2^Department of Medicine Huddinge, Karolinska Institute, Stockholm, Sweden. ^3^Department of Infectious Diseases, Karolinska University Hospital, Stockholm, Sweden. ^4^Department of Emergency Medicine, Karolinska University Hospital, Stockholm, Sweden. ^5^Department of Perioperative Medicine and Intensive Care, Karolinska University Hospital, Stockholm, Sweden. ^6^The Laboratory for Molecular Infection Medicine Sweden, Umeå, Sweden. ^7^Department of Clinical Pathology and Cancer Diagnostics, Karolinska University Hospital, Stockholm, Sweden. ^8^Childhood Cancer Research Unit, Department of Women’s and Children’s Health, Karolinska Institutet, Stockholm, Sweden. ^9^Theme Aging, Karolinska University Hospital, Stockholm, Sweden. ^10^Department of Clinical Interventions and Technology CLINTEC, Division for Anesthesiology and Intensive Care, Karolinska Institutet, Stockholm, Sweden

## Supplementary Material

Supplemental 1 (PDF)

## References

[1] Merad, M., C. A. Blish, F. Sallusto, A. Iwasaki. 2022. The immunology and immunopathology of COVID-19. Science 375: 1122–1127. 35271343 10.1126/science.abm8108 PMC12828912

[2] Lamers, M. M., B. L. Haagmans. 2022. SARS-CoV-2 pathogenesis. Nat. Rev. Microbiol. 20: 270–284. 35354968 10.1038/s41579-022-00713-0

[3] Ahamed, J., J. Laurence. 2022. Long COVID endotheliopathy: hypothesized mechanisms and potential therapeutic approaches. J. Clin. Invest. 132: e161167. 35912863 10.1172/JCI161167 PMC9337829

[4] Al-Aly, Z., Y. Xie, B. Bowe. 2021. High-dimensional characterization of post-acute sequelae of COVID-19. Nature 594: 259–264. 33887749 10.1038/s41586-021-03553-9

[5] Sette, A., S. Crotty. 2022. Immunological memory to SARS-CoV-2 infection and COVID-19 vaccines. Immunol. Rev. 310: 27–46. 35733376 10.1111/imr.13089 PMC9349657

[6] Blanco-Melo, D., B. E. Nilsson-Payant, W. C. Liu, S. Uhl, D. Hoagland, R. Møller, T. X. Jordan, K. Oishi, M. Panis, D. Sachs, . 2020. Imbalanced host response to SARS-CoV-2 drives development of COVID-19. Cell 181: 1036–1045.e9. 32416070 10.1016/j.cell.2020.04.026 PMC7227586

[7] Ziegler, C. G. K., V. N. Miao, A. H. Owings, A. W. Navia, Y. Tang, J. D. Bromley, P. Lotfy, M. Sloan, H. Laird, H. B. Williams, . 2021. Impaired local intrinsic immunity to SARS-CoV-2 infection in severe COVID-19. Cell 184: 4713–4733.e22. 34352228 10.1016/j.cell.2021.07.023 PMC8299217

[8] Hadjadj, J., N. Yatim, L. Barnabei, A. Corneau, J. Boussier, N. Smith, H. Péré, B. Charbit, V. Bondet, C. Chenevier-Gobeaux, . 2020. Impaired type I interferon activity and inflammatory responses in severe COVID-19 patients. Science 369: 718–724. 32661059 10.1126/science.abc6027 PMC7402632

[9] Schulte-Schrepping, J., N. Reusch, D. Paclik, K. Baßler, S. Schlickeiser, B. Zhang, B. Krämer, T. Krammer, S. Brumhard, L. Bonaguro, Deutsche COVID-19 OMICS Initiative (DeCOI). 2020. Severe COVID-19 is marked by a dysregulated myeloid cell compartment. Cell 182: 1419–1440.e23. 32810438 10.1016/j.cell.2020.08.001 PMC7405822

[10] Parrot, T., J. B. Gorin, A. Ponzetta, K. T. Maleki, T. Kammann, J. Emgard, A. Perez-Potti, T. Sekine, O. Rivera-Ballesteros, C.-S. G. Karolinska, . 2020. MAIT cell activation and dynamics associated with COVID-19 disease severity. Sci. Immunol. 5: eabe1670. 32989174 10.1126/sciimmunol.abe1670 PMC7857393

[11] Flament, H., M. Rouland, L. Beaudoin, A. Toubal, L. Bertrand, S. Lebourgeois, C. Rousseau, P. Soulard, Z. Gouda, L. Cagninacci, . 2021. Outcome of SARS-CoV-2 infection is linked to MAIT cell activation and cytotoxicity. Nat. Immunol. 22: 322–335. 33531712 10.1038/s41590-021-00870-z

[12] Jouan, Y., A. Guillon, L. Gonzalez, Y. Perez, C. Boisseau, S. Ehrmann, M. Ferreira, T. Daix, R. Jeannet, B. François, . 2020. Phenotypical and functional alteration of unconventional T cells in severe COVID-19 patients. J. Exp. Med. 217: e20200872. 32886755 10.1084/jem.20200872 PMC7472174

[13] Kjer-Nielsen, L., O. Patel, A. J. Corbett, J. Le Nours, B. Meehan, L. Liu, M. Bhati, Z. Chen, L. Kostenko, R. Reantragoon, . 2012. MR1 presents microbial vitamin B metabolites to MAIT cells. Nature 491: 717–723. 23051753 10.1038/nature11605

[14] Corbett, A. J., S. B. Eckle, R. W. Birkinshaw, L. Liu, O. Patel, J. Mahony, Z. Chen, R. Reantragoon, B. Meehan, H. Cao, . 2014. T-cell activation by transitory neo-antigens derived from distinct microbial pathways. Nature 509: 361–365. 24695216 10.1038/nature13160

[15] Boudinot, P., S. Mondot, L. Jouneau, L. Teyton, M. P. Lefranc, O. Lantz. 2016. Restricting nonclassical MHC genes coevolve with TRAV genes used by innate-like T cells in mammals. Proc. Natl. Acad. Sci. USA 113: E2983–E2992. 27170188 10.1073/pnas.1600674113 PMC4889381

[16] Mak, J. Y. W., L. Liu, D. P. Fairlie. 2021. Chemical modulators of mucosal associated invariant T cells. Acc. Chem. Res. 54: 3462–3475. 34415738 10.1021/acs.accounts.1c00359 PMC8989627

[17] Provine, N. M., P. Klenerman. 2020. MAIT cells in health and disease. Annu. Rev. Immunol. 38: 203–228. 31986071 10.1146/annurev-immunol-080719-015428

[18] Nel, I., L. Bertrand, A. Toubal, A. Lehuen. 2021. MAIT cells, guardians of skin and mucosa? Mucosal Immunol. 14: 803–814. 33753874 10.1038/s41385-021-00391-w PMC7983967

[19] Meierovics, A., W. J. Yankelevich, S. C. Cowley. 2013. MAIT cells are critical for optimal mucosal immune responses during in vivo pulmonary bacterial infection. Proc. Natl. Acad. Sci. USA 110: E3119–E3128. 23898209 10.1073/pnas.1302799110 PMC3746930

[20] Wang, H., C. D’Souza, X. Y. Lim, L. Kostenko, T. J. Pediongco, S. B. G. Eckle, B. S. Meehan, M. Shi, N. Wang, S. Li, . 2018. MAIT cells protect against pulmonary *Legionella longbeachae* infection. Nat. Commun. 9: 3350. 30135490 10.1038/s41467-018-05202-8 PMC6105587

[21] Georgel, P., M. Radosavljevic, C. Macquin, S. Bahram. 2011. The non-conventional MHC class I MR1 molecule controls infection by *Klebsiella pneumoniae* in mice. Mol. Immunol. 48: 769–775. 21190736 10.1016/j.molimm.2010.12.002

[22] Dusseaux, M., E. Martin, N. Serriari, I. Péguillet, V. Premel, D. Louis, M. Milder, L. Le Bourhis, C. Soudais, E. Treiner, O. Lantz. 2011. Human MAIT cells are xenobiotic-resistant, tissue-targeted, CD161^hi^ IL-17-secreting T cells. Blood 117: 1250–1259. 21084709 10.1182/blood-2010-08-303339

[23] Dias, J., E. Leeansyah, J. K. Sandberg. 2017. Multiple layers of heterogeneity and subset diversity in human MAIT cell responses to distinct microorganisms and to innate cytokines. Proc. Natl. Acad. Sci. USA 114: E5434–E5443. 28630305 10.1073/pnas.1705759114 PMC5502643

[24] Kurioka, A., J. E. Ussher, C. Cosgrove, C. Clough, J. R. Fergusson, K. Smith, Y. H. Kang, L. J. Walker, T. H. Hansen, C. B. Willberg, P. Klenerman. 2015. MAIT cells are licensed through granzyme exchange to kill bacterially sensitized targets. Mucosal Immunol. 8: 429–440. 25269706 10.1038/mi.2014.81 PMC4288950

[25] Boulouis, C., W. R. Sia, M. Y. Gulam, J. Q. M. Teo, Y. T. Png, T. K. Phan, J. Y. W. Mak, D. P. Fairlie, I. K. H. Poon, T. H. Koh, . 2020. Human MAIT cell cytolytic effector proteins synergize to overcome carbapenem resistance in *Escherichia coli*. PLoS Biol. 18: e3000644. 32511236 10.1371/journal.pbio.3000644 PMC7302869

[26] Constantinides, M. G., V. M. Link, S. Tamoutounour, A. C. Wong, P. J. Perez-Chaparro, S. J. Han, Y. E. Chen, K. Li, S. Farhat, A. Weckel, . 2019. MAIT cells are imprinted by the microbiota in early life and promote tissue repair. Science 366: eaax6624. 31649166 10.1126/science.aax6624 PMC7603427

[27] Hinks, T. S. C., E. Marchi, M. Jabeen, M. Olshansky, A. Kurioka, T. J. Pediongco, B. S. Meehan, L. Kostenko, S. J. Turner, A. J. Corbett, . 2019. Activation and in vivo evolution of the MAIT cell transcriptome in mice and humans reveals tissue repair functionality. Cell Rep. 28: 3249–3262.e5. 31533045 10.1016/j.celrep.2019.07.039 PMC6859474

[28] Leng, T., H. D. Akther, C. P. Hackstein, K. Powell, T. King, M. Friedrich, Z. Christoforidou, S. McCuaig, M. Neyazi, C. V. Arancibia-Cárcamo, Oxford IBD Investigators. 2019. TCR and inflammatory signals tune human MAIT cells to exert specific tissue repair and effector functions. Cell Rep. 28: 3077–3091.e5. 31533032 10.1016/j.celrep.2019.08.050 PMC6899450

[29] van Wilgenburg, B., I. Scherwitzl, E. C. Hutchinson, T. Leng, A. Kurioka, C. Kulicke, C. de Lara, S. Cole, S. Vasanawathana, W. Limpitikul, STOP-HCV consortium. 2016. MAIT cells are activated during human viral infections. Nat. Commun. 7: 11653. 27337592 10.1038/ncomms11653 PMC4931007

[30] Long, Y., T. S. C. Hinks. 2021. MAIT cells in respiratory viral infections in mouse and human. Crit. Rev. Immunol. 41: 19–35. 35381137 10.1615/CritRevImmunol.2021040877 PMC7612767

[31] Han, F., Y. Zheng, A. Ho, S. Ma, J. K. Sandberg, E. Leeansyah. 2021. MAIT cell loss and reconstitution in HIV-1 disease. Crit. Rev. Immunol. 41: 69–82. 10.1615/CritRevImmunol.2022042906 36047323

[32] Sandberg, J. K., E. Leeansyah, M. A. Eller, B. L. Shacklett, D. Paquin-Proulx. 2023. The emerging role of MAIT cell responses in viral infections. J. Immunol. 211: 511–517. 37549397 10.4049/jimmunol.2300147 PMC10421619

[33] van Wilgenburg, B., L. Loh, Z. Chen, T. J. Pediongco, H. Wang, M. Shi, Z. Zhao, M. Koutsakos, S. Nüssing, S. Sant, . 2018. MAIT cells contribute to protection against lethal influenza infection in vivo. Nat. Commun. 9: 4706. 30413689 10.1038/s41467-018-07207-9 PMC6226485

[34] Loh, L., Z. Wang, S. Sant, M. Koutsakos, S. Jegaskanda, A. J. Corbett, L. Liu, D. P. Fairlie, J. Crowe, J. Rossjohn, . 2016. Human mucosal-associated invariant T cells contribute to antiviral influenza immunity via IL-18-dependent activation. Proc. Natl. Acad. Sci. USA 113: 10133–10138. 27543331 10.1073/pnas.1610750113 PMC5018778

[35] Lamichhane, R., M. Schneider, S. M. de la Harpe, T. W. R. Harrop, R. F. Hannaway, P. K. Dearden, J. R. Kirman, J. D. A. Tyndall, A. J. Vernall, J. E. Ussher. 2019. TCR- or cytokine-activated CD8^+^ mucosal-associated invariant T cells are rapid polyfunctional effectors that can coordinate immune responses. Cell Rep. 28: 3061–3076.e5. 31533031 10.1016/j.celrep.2019.08.054

[36] Maleki, K. T., J. Tauriainen, M. García, P. F. Kerkman, W. Christ, J. Dias, J. Wigren Byström, E. Leeansyah, M. N. Forsell, H. G. Ljunggren, . 2021. MAIT cell activation is associated with disease severity markers in acute hantavirus infection. Cell Rep. Med. 2: 100220. 33763658 10.1016/j.xcrm.2021.100220 PMC7974553

[37] Youngs, J., N. M. Provine, N. Lim, H. R. Sharpe, A. Amini, Y. L. Chen, J. Luo, M. D. Edmans, P. Zacharopoulou, W. Chen, Oxford Protective T cell Immunology for COVID-19 (OPTIC) Clinical team. 2021. Identification of immune correlates of fatal outcomes in critically ill COVID-19 patients. PLoS Pathog. 17: e1009804. 34529726 10.1371/journal.ppat.1009804 PMC8445447

[38] Sekine, T., A. Perez-Potti, O. Rivera-Ballesteros, K. Strålin, J. B. Gorin, A. Olsson, S. Llewellyn-Lacey, H. Kamal, G. Bogdanovic, S. Muschiol, Karolinska COVID-19 Study Group. 2020. Robust T cell immunity in convalescent individuals with asymptomatic or mild COVID-19. Cell 183: 158–168.e14. 32979941 10.1016/j.cell.2020.08.017 PMC7427556

[39] Maucourant, C., I. Filipovic, A. Ponzetta, S. Aleman, M. Cornillet, L. Hertwig, B. Strunz, A. Lentini, B. Reinius, D. Brownlie, Karolinska COVID-19 Study Group. 2020. Natural killer cell immunotypes related to COVID-19 disease severity. Sci. Immunol. 5: eabd6832. 32826343 10.1126/sciimmunol.abd6832 PMC7665314

[40] Ljunggren, H. G., E. H. Ask, M. Cornillet, B. Strunz, P. Chen, J. Rao Muvva, M. Akber, M. Buggert, B. J. Chambers, A. Cuapio, Karolinska KI/K COVID-19 Study Group. 2022. The Karolinska KI/K COVID-19 Immune Atlas: an open resource for immunological research and educational purposes. Scand. J. Immunol. 96: e13195. 35652743 10.1111/sji.13195 PMC9287045

[41] Mak, J. Y., W. Xu, R. C. Reid, A. J. Corbett, B. S. Meehan, H. Wang, Z. Chen, J. Rossjohn, J. McCluskey, L. Liu, D. P. Fairlie. 2017. Stabilizing short-lived Schiff base derivatives of 5-aminouracils that activate mucosal-associated invariant T cells. Nat. Commun. 8: 14599. 28272391 10.1038/ncomms14599 PMC5344979

[42] Lin, L., G. Finak, K. Ushey, C. Seshadri, T. R. Hawn, N. Frahm, T. J. Scriba, H. Mahomed, W. Hanekom, P. A. Bart, . 2015. COMPASS identifies T-cell subsets correlated with clinical outcomes. Nat. Biotechnol. 33: 610–616. 26006008 10.1038/nbt.3187 PMC4569006

[43] Strunz, B., J. Hengst, K. Deterding, M. P. Manns, M. Cornberg, H. G. Ljunggren, H. Wedemeyer, N. K. Björkström. 2018. Chronic hepatitis C virus infection irreversibly impacts human natural killer cell repertoire diversity. Nat. Commun. 9: 2275. 29891939 10.1038/s41467-018-04685-9 PMC5995831

[44] Yang, Q., Y. Wen, F. Qi, X. Gao, W. Chen, G. Xu, C. Wei, H. Wang, X. Tang, J. Lin, . 2021. Suppressive monocytes impair MAIT cells response via IL-10 in patients with severe COVID-19. J. Immunol. 207: 1848–1856. 34452933 10.4049/jimmunol.2100228

[45] Shi, J., J. Zhou, X. Zhang, W. Hu, J. F. Zhao, S. Wang, F. S. Wang, J. Y. Zhang. 2021. Single-cell transcriptomic profiling of MAIT cells in patients with COVID-19. Front. Immunol. 12: 700152. 34394094 10.3389/fimmu.2021.700152 PMC8363247

[46] Lal, K. G., D. Kim, M. C. Costanzo, M. Creegan, E. Leeansyah, J. Dias, D. Paquin-Proulx, L. A. Eller, A. Schuetz, Y. Phuang-Ngern, . 2020. Dynamic MAIT cell response with progressively enhanced innateness during acute HIV-1 infection. Nat. Commun. 11: 272. 31937782 10.1038/s41467-019-13975-9 PMC6959336

[47] Leeansyah, E., A. Ganesh, M. F. Quigley, A. Sönnerborg, J. Andersson, P. W. Hunt, M. Somsouk, S. G. Deeks, J. N. Martin, M. Moll, . 2013. Activation, exhaustion, and persistent decline of the antimicrobial MR1-restricted MAIT-cell population in chronic HIV-1 infection. Blood 121: 1124–1135. 23243281 10.1182/blood-2012-07-445429 PMC3575756

[48] Cosgrove, C., J. E. Ussher, A. Rauch, K. Gärtner, A. Kurioka, M. H. Hühn, K. Adelmann, Y. H. Kang, J. R. Fergusson, P. Simmonds, . 2013. Early and nonreversible decrease of CD161^++^/MAIT cells in HIV infection. Blood 121: 951–961. 23255555 10.1182/blood-2012-06-436436 PMC3567342

[49] Dias, J., J. Hengst, T. Parrot, E. Leeansyah, S. Lunemann, D. F. G. Malone, S. Hardtke, O. Strauss, C. L. Zimmer, L. Berglin, . 2019. Chronic hepatitis delta virus infection leads to functional impairment and severe loss of MAIT cells. J. Hepatol. 71: 301–312. 31100314 10.1016/j.jhep.2019.04.009 PMC6642010

[50] Provine, N. M., A. Amini, L. C. Garner, A. J. Spencer, C. Dold, C. Hutchings, L. Silva Reyes, M. E. B. FitzPatrick, S. Chinnakannan, B. Oguti, . 2021. MAIT cell activation augments adenovirus vector vaccine immunogenicity. Science 371: 521–526. 33510029 10.1126/science.aax8819 PMC7610941

[51] Boulouis, C., T. Kammann, A. Cuapio, T. Parrot, Y. Gao, E. Mouchtaridi, D. Wullimann, J. Lange, P. Chen, M. Akber, COVAXID study group. 2022. MAIT cell compartment characteristics are associated with the immune response magnitude to the BNT162b2 mRNA anti-SARS-CoV-2 vaccine. Mol. Med. 28: 54. 35562666 10.1186/s10020-022-00484-7 PMC9100314

[52] Bister, J., Y. Crona Guterstam, B. Strunz, B. Dumitrescu, K. Haij Bhattarai, V. Özenci, M. Brännström, M. A. Ivarsson, S. Gidlöf, N. K. Björkström. 2021. Human endometrial MAIT cells are transiently tissue resident and respond to *Neisseria* gonorrhoeae. Mucosal Immunol. 14: 357–365. 32759973 10.1038/s41385-020-0331-5

