# Supplementary material for: Dynamic MAIT Cell Recovery after Severe COVID-19 Is Transient with Signs of Heterogeneous Functional Anomalies
Source: J Immunol. 2023 Dec 20;212(3):389–96. doi: 10.4049/jimmunol.2300639 (PMC10784727; doi:10.4049/jimmunol.2300639)
Supplement: Supplemental 1 (PDF) [file JI_2300639_Supplemental_1.pdf]

Supplementary Table I. Demographic and clinical characteristics from the acute stage of the COVID-19 patients included for follow-up at 4-5 months, and 9 months post infection.

		**Healthy donors**	**COVID-19**
**n**		11	16
**Age group (years)**	34-49	27% (3)	31% (5)
	50-59	55% (6)	31% (5)
	60-69	18% (2)	31% (5)
	70-79	0% (0)	5% (1)
**Gender**	Male	73% (8)	81% (13)
	Female	27% (3)	19% (3)
**BMI (median; IQR)**		nd	30 ± 8.6
**Disease severity**	Moderate-severe	0%	50% (8)
	Severe	0%	50% (8)
**Smoking**	No	nd	31% (5)
	Past	nd	13% (2)
	Yes	nd	25% (4)
	Unknown	nd	31% (5)
**Comorbidities**	Hypertension	nd	31% (5)
	Diabetes	nd	31% (5)
	Cardiovascular disease	nd	13% (2)
**Positive viremia before acute sampling**		nd	38% (6)
**Positive blood culture +/- 5 d before acute sampling**		nd	13% (2)
**Positive lower resp culture +/- 5 d before acute sampling**		nd	25% (4)
**SOFA total before acute sampling**	1	nd	44% (7)
	2	nd	6% (1)
	3	nd	31% (5)
	4	nd	6% (1)
	7	nd	12% (2)
**Oxygen treatment before acute sampling**	Low flow < 15 L/min	0%	37% (6)
	High flow > 15 L/min	0%	25% (4)
	Ventilator	0%	37% (6)
**Steroids prior to acute sampling**		nd	37% (6)
**Antibiotics prior to acute sampling**		nd	44% (7)
**CMV IgG prior to acute sampling**		nd	94% (15)
**SARS-CoV-2 IgG prior to acute sampling**		0%	69% (11)

BMI: body mass index, GI: Gastrointestinal, CMV: cytomegalovirus, nd: non-determined, IQR: interquartile range.

Supplementary Table II. Demographic and clinical characteristics from a second group of COVID-19 patients sampled at four to five months convalescent cross-sectional follow-up.

	**COVID-19**
**n**	24
**Age group (years)**	
18–33	3 (13%)
34–49	5 (21%)
50–59	9 (38%)
60–69	7 (29%)
70–79	0 (0%)
**Gender**	
Male	17 (71%)
Female	7 (29%)
**BMI (median; IQR)**	28.4 ± 7.1
**Disease severity**	
Moderate-severe	0% (0)
Severe (ICU)	100% (24)
**Comorbidities**	
Hypertension	50% (12)
Diabetes	13% (3)
Cardiovascular disease	17% (4)

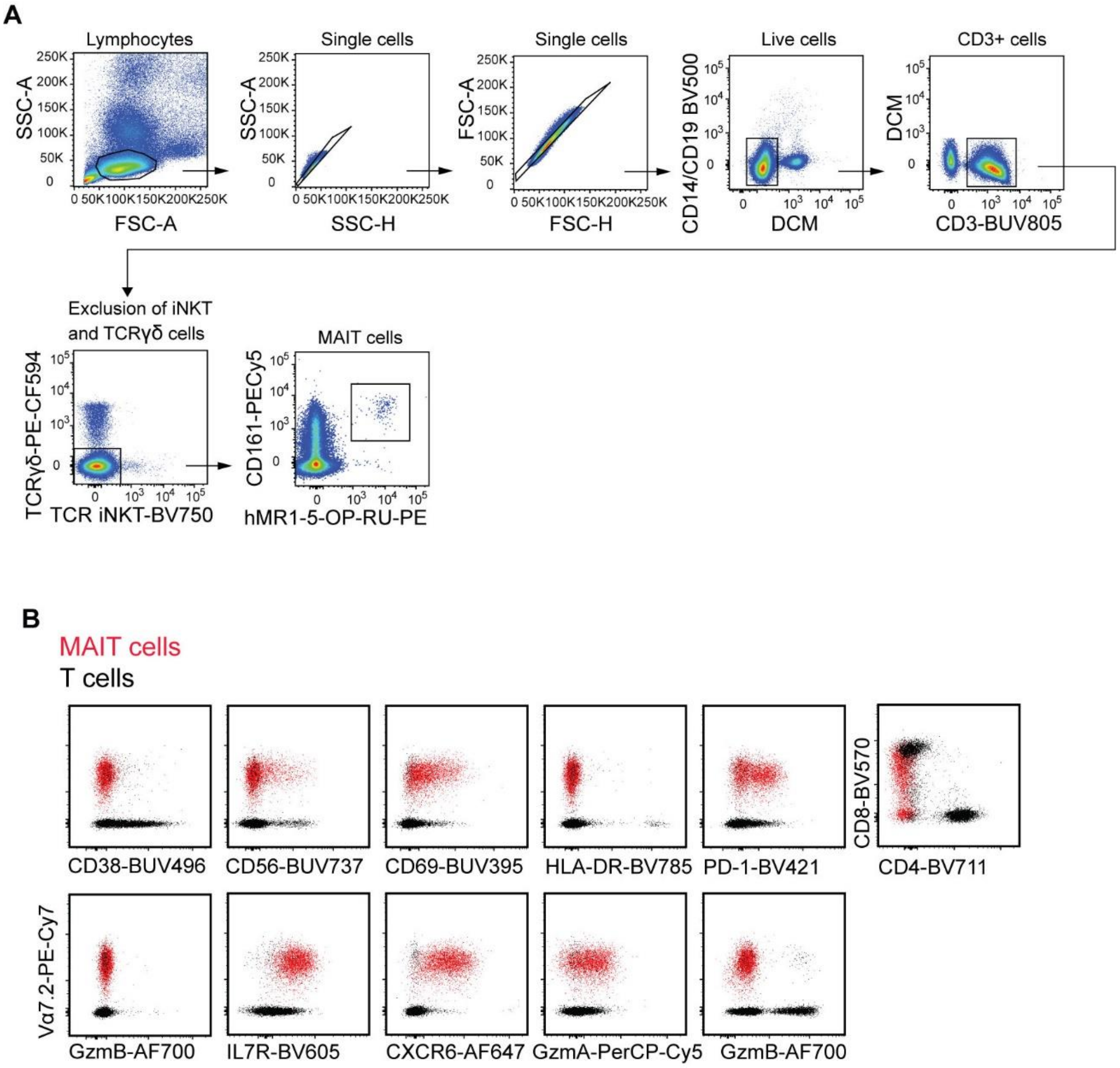

Supplementary Figure 1. Gating strategy for the phenotypic analysis of MAIT cells. (A) Representative gating strategy for the identification of MAIT cells. Prior to the gating, data were cleaned using the FlowAI tool that checks for flow stability over time. (B) Representative FACS plots for the gating of CD4, CD8, CD56, CD69, CD38, HLA-DR, Granzyme A, Granzyme B, PD-1, IL-7R, and CXCR6 on MAIT cells. The non-MAIT $CD3^+$ T cell population in black were used as a reference to set the gates.

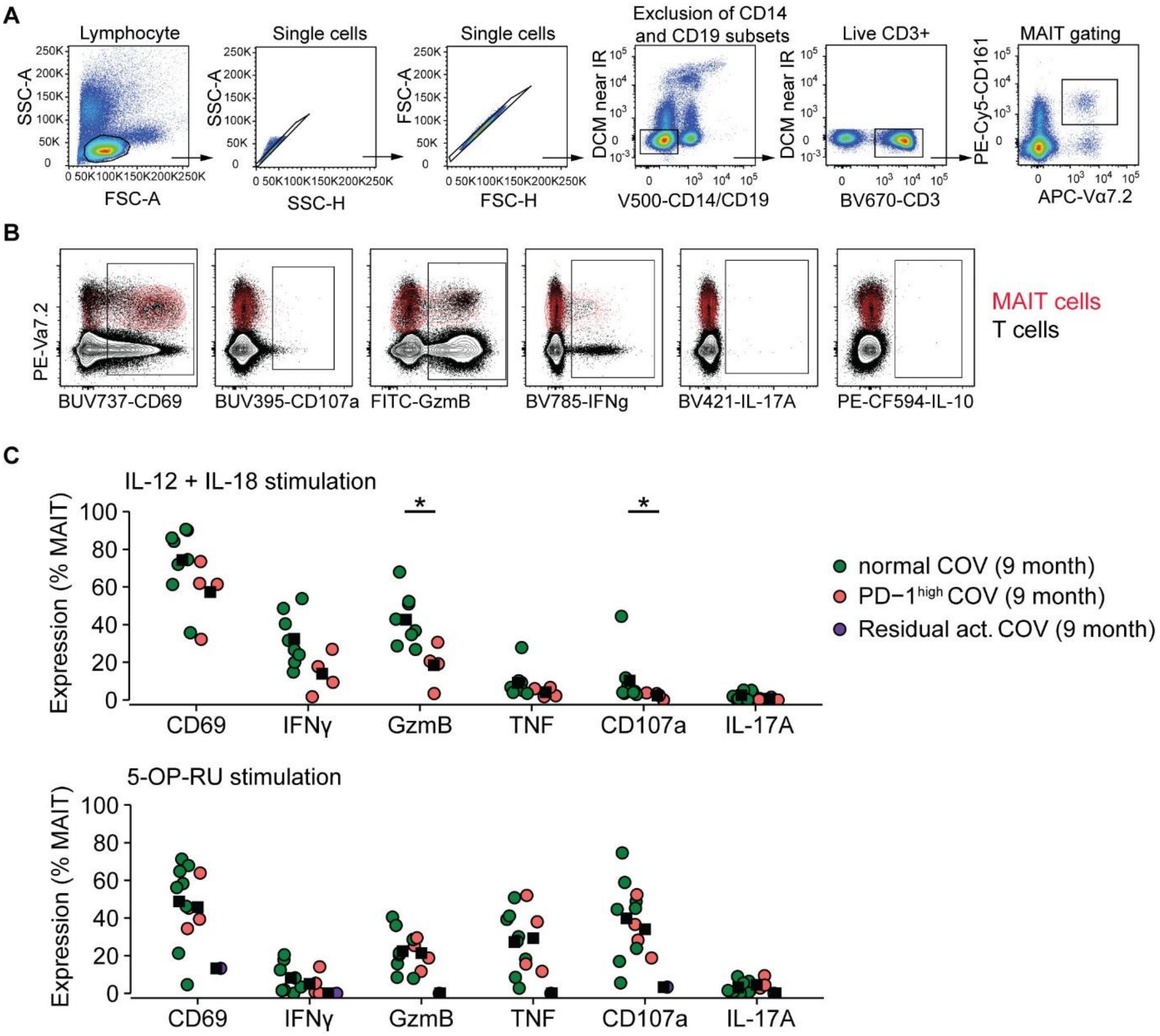

Supplementary Figure 2. Gating strategy for the functional analysis of MAIT cells. (A) Representative gating strategy for the identification of MAIT cells. Prior to the gating, data were cleaned using the FlowAI tool that checks for flow stability over time. MAIT cells were gated using CD161 in combination with intracellular staining of Vα7.2. (B) Representative FACS plots for the gating of CD69, CD107a, GzmB, IFNγ, TNF, IL-17A and IL-10 in MAIT cells. The non-MAIT $CD3^+$ T cells in black were used as a reference to set the gates. (C) Relative expression of activation, cytokine and effector markers expressed on MAIT cells at the nine-month convalescent phase following IL-12+IL-18 stimulation (top) and 5-OP-RU stimulation (bottom). *$p<0.05$.
